# Genetics of female and male infertility

**DOI:** 10.1515/medgen-2024-2040

**Published:** 2024-09-06

**Authors:** Corinna Friedrich, Frank Tüttelmann

**Affiliations:** University and University Hospital Münster Centre of Medical Genetics, Institute of Reproductive Genetics Vesaliusweg 12–14 48149 Münster Germany; University and University Hospital Münster Centre of Medical Genetics, Institute of Reproductive Genetics Vesaliusweg 12–14 48149 Münster Germany

**Keywords:** couple infertility, genetic diagnostics, genetic counselling, medically assisted reproduction (MAR)

## Abstract

Infertility is defined as the inability to conceive within one year of unprotected intercourse, and the causes are equally distributed between both sexes. Genetics play a crucial role in couple infertility and respective diagnostic testing should follow available guidelines. Appropriate tiered genetic analyses require comprehensive physical examination of both partners in an infertile couple. A wide range of chromosomal and monogenic variants can be the underlying genetic cause of infertility in both women and men. Accurate clinical phenotyping, together with identification of the genetic origin, helps to recommend the proper treatment and to counsel couples on the success rates and potential risks for offspring.

## Introduction/background

In his foreword of the recent report *Infertility prevalence estimates, 1990–2021*, Dr. Tedros Adhanom Ghebreyesus, Director-General of the World Health Organisation (WHO), states that the access to reproductive health including infertility treatment is a right independent from ethnical, social, or national origin [27]. To achieve this right, it is required to understand infertility’s prevalence, the causes involved, and their impact on clinical decision making. According to the WHO, infertility is defined as a couples’ inability to conceive after one year of regular unprotected intercourse, and one in six people worldwide, men and women, is affected by infertility during their lifetime. The phenotypes and causes of couple infertility are highly diverse. As in other complex diseases, genetics likely play a major role, but the molecular mechanisms and associated genetic causes remain poorly understood. Therefore, although a genetic origin is frequently suspected, the diagnostic yield of the standard genetic analyses is only 10–20 % in men and 5–10 % in women [30]. In Germany, the AWMF S2k guideline 015/085 addressed the examination and genetic diagnostics of infertile couples before medically assisted reproduction (MAR), which is the only option to conceive a child in most cases. This guideline is the basis of this review [11].

## Tiered genetic diagnostics for male infertility

**Figure 1:**
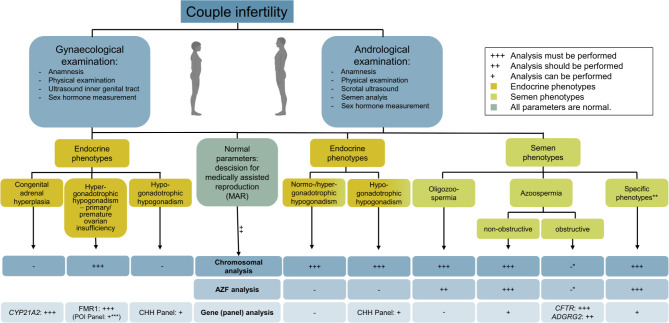
Tiered genetic diagnostics in couple infertility according to AWMF guideline 015/085. *Because the definitive diagnosis of CBAVD can only be made during the surgery and not only based on hormonal and clinical parameters, the chromosomal and AZF analyses should be performed in case of an inconclusive clinical diagnosis. **Specific phenotypes such as multiple morphological abnormalities of sperm flagella (MMAF) or forms of astheno-/teratozoospermia are often accompanied by oligozoospermia and, thus, karyotype and AZF analysis are indicated. ***Due to recent advances in exome sequencing, a gene panel analyses could be performed in cases of unexplained primary/premature ovarian insufficiency (POI).

In the case of couple infertility and prior to MAR treatment, the male partner must undergo andrological examination. This includes anamnesis, physical examination, scrotal ultrasound, semen analysis, and determination of sex hormones. After exclusion of non-genetic factors, e. g. oncological diseases, chemo-/radiotherapy, or infections, men can be categorised into those with abnormal semen parameters and those with endocrinological phenotypes, thus the indicated genetic testing strategy can be established as outlined in Figure 1.

### Chromosomal aberrations

Those men with oligozoospermia (<39 million total sperm count, <15 million/mL sperm concentration) or azoospermia (no sperm found in the semen sample) a chromosomal analysis must be offered.

The most frequent genetic cause of azoospermia and (rarely) oligozoospermia is the Klinefelter syndrome, karyotype 47,XXY, that explains the azoospermia in up to 15 % of affected men [29]. Klinefelter syndrome is a rather common *de novo* chromosomal aberration with a prevalence of 1/500 to 1/700 in male newborns [5, 7]. It is estimated that only 25 % of affected individuals are diagnosed during their lifetime. In many cases, men with a 47,XXY karyotype receive their diagnosis only during evaluation for infertility. The sperm retrieval rate via testicular sperm extraction (TESE) for subsequent intracytoplasmic sperm injection (ICSI) is around 30–50 %, depending on the age of the patients and surgical approaches [5]. If men with Klinefelter syndrome father children, the risk for aneuploidies in the offspring is not increased. Hormonal treatment will not improve fertility in Klinefelter syndrome and MAR is the treatment of choice to achieve a pregnancy of the partner. Of note, testosterone treatment might further impair the residual spermatogenesis in affected men. A testicular biopsy/TESE for fertility preservation should likely be offered as early as possible and before starting testosterone substitution [19].

In up to 5 % of men with azoospermia, other chromosomal aberrations are identified [29]. These include aberrations of the sex chromosomes such as a karyotype of 46,XX in phenotypic males (in >90 % caused by a translocation of the sex determining region of Y (*SRY*) gene) and derivative Y-chromosomes. The latter can be seen as a ring chromosome or an isochromosome with the need to analyse for the presence or absence of the azoospermia factor (AZF) regions. In addition, balanced structural aberrations, mostly translocations, are associated with reduced sperm counts, i. e., have a significantly higher prevalence in men with oligo-/azoospermia [4]. However, in contrast to Klinefelter syndrome and sex-chromosomal aberrations, autosomal translocations do not *per se* constitute a causal diagnosis for impaired spermatogenesis because these aberrations are frequently also found in fertile family members. Importantly, structural chromosomal aberrations pose a significant risk for unbalanced sperm and, thus, aneuploid embryos, nonviable pregnancies, miscarriages, or birth of children with malformations or developmental disorder. Thus, a chromosomal analysis is also indicated in couples with recurrent pregnancy loss. Affected couples must be counselled about the mentioned risks and the possibility for prenatal and preimplantation genetic testing (PND/PGT).

### Microdeletions of the azoospermia factor (AZF) region

In addition to a chromosomal analysis, men with azoospermia must and men with oligozoospermia should be offered an analysis of the Y-chromosomal AZF region [10]. Microdeletions of the AZF region account for up to 4 % of genetic diagnosis in men with azoospermia [29]. The AZF region comprises the three segments AZFa, AZFb, and AZFc. The AZFc deletion is the most frequent type (70–80 %) and the TESE success rate is relatively high with around 50 % [10]. Microdeletions of the AZFa (0.5–9 %), AZFb (1–7 %), and AZFbc (1–20 %) region are less frequent and, here, the TESE success rates are virtually zero. The affected patients must be counselled about the inheritance of the Y-chromosomal AZF deletion, and thus, the infertility to their sons.

**Figure 2:**
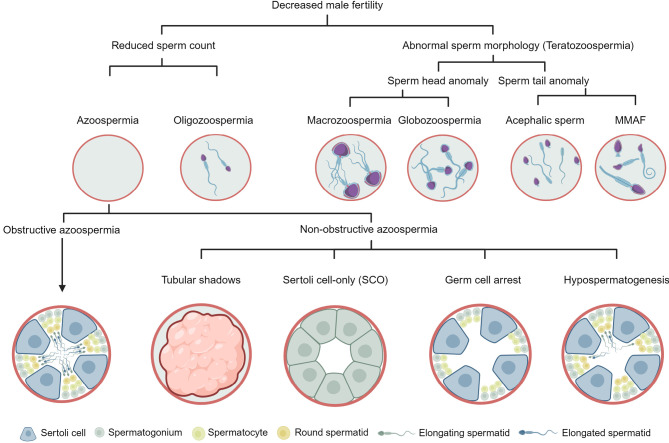
**Different phenotypes of male infertility.** In case of couple infertility, the male partner undergoes andrological examination including semen analysis. The semen sample is evaluated with regard to sperm count, morphology, and motility. Based on these parameters, different phenotypes are defined. MMAF: multiple morphological abnormalities of the sperm flagella. Modified from [28].

### Monogenic causes of spermatogenic failure

Most failures during the process of sperm production (spermatogenesis) have an impact on sperm count, sperm morphology/structure, and/or motility and can typically be detected in the semen sample. For these phenotypes, monogenic factors are (suspected to be) a likely cause. To identify the causal genetic variant, sequence analysis of genes with sufficient (at least moderate) clinical evidence for a specific phenotype according to clinical guidelines established by the Clinical Genome Resource (ClinGen) and the American College of Medical Genetics (ACMG) can/must be performed. Most of the monogenic spermatogenic failures described so far follow an autosomal recessive inheritance but some X- or Y-linked as well as autosomal dominant variants are also relevant as summarised in Table 1.

For further genetic diagnostics of men with azoospermia, it is important to distinguish between obstructive and non-obstructive forms (Figure 2).

### Obstructive azoospermia

The testicular phenotype of obstructive azoospermia (OA; HP:0011962) is characterised by complete spermatogenesis (Figure 2) and, thus, the TESE success rate is almost 100 % [20]. Obstructive azoospermia is often but not necessarily caused by the *congenital absence of the vas deferens* (CBAVD). In more than 80 % of patients it can be regarded as a minimal form of cystic fibrosis caused by biallelic *CFTR* pathogenic variants. In approximately 2 % of cases with CBAVD, pathogenic hemizygous loss-of-function variants are found in the *ADGRG2* gene. Accordingly, when CBAVD is suspected because specific semen parameters in the presence of normal testicular volume and follicle stimulating hormone (FSH) serum levels, the analysis of the genes *CFTR* and* ADGRG2* is indicated. The diagnostic yield of *CFTR* and *ADGRG2* analysis is around 2 % considering all cases with azoospermia [29]. If pathogenic variants are identified, standard procedures apply such as genetic counselling and, especially in case of *CFTR-*related azoospermia*,* analysis of the female partner to assess the risk for cystic fibrosis in offspring.

### Non-obstructive Azoospermia

In contrast to obstructive azoospermia, in non-obstructive azoospermia (NOA; HP:0011961), spermatogenesis is impaired at different stages [15, 28]. Therefore, non-obstructive azoospermia is characterised by different testicular histological phenotypes that range from spermiation failure with full spermatogenesis (HP:0034812), hypospermatogenesis (HP:0034813), germ cell arrest (HP:0031038), Sertoli cell-only (SCO, HP:0034299), to the most severe form of tubular shadows (HP:0034945).

In spermiation failure, the histology is characterised by complete spermatogenesis but the release of elongated spermatids into the lumen of the germinal tubules is impaired. Hypospermatogenesis is histologically characterised by complete but quantitatively reduced spermatogenesis. Germ cell arrest describes a block of spermatogenesis at any stage and can be specified as spermatogonial arrest (HP:4000187), spermatocyte/meiotic arrest (HP:0031039), and round spermatid arrest (HP:0031040). In the SCO phenotype, there are no germ cells and only somatic Sertoli cells present. The most severe histological phenotype are tubular shadows, which are characterised by testicular atrophy resulting from the loss of germ and Sertoli cells, thickening of the basal lamina, and hyalinisation. These phenotypes manifest in a focal, predominant, or complete manner, and can occur similarly or differently distributed in both testes.

In the semen sample, all of the described testicular phenotypes result in azoospermia or cryptozoospermia (HP:0030974), which is a subform of extreme oligozoospermia (HP:0034815). In cryptozoospermia, very few sperm are only found after centrifugation. In around 4 % of men with crypto-/azoospermia, a monogenic cause can be identified when analysing a clinically validated gene panel comprising 20 genes [29]. In parallel to the constantly increasing number of validated genes (now already 36 as reviewed in [23], Table 1), the diagnostic yield also increases continuously. The TESE success rate varies significantly and depends on the testicular phenotype that is a result of the underlying gene defect. While for most validated genes the TESE success is virtually zero, a few cases of positive TESE outcome have been described for some genes such as *M1AP* and *TEX15* [29]. To date, comprehensive genetic analyses in large cohorts of patients with spermatogenic failure are lacking, precluding firm conclusions and evidence-based treatment decisions.

### Abnormal sperm motility and/or morphology

Besides sperm count, the semen sample is also analysed for sperm motility and morphology (Figure 2). When motility is impaired, this condition is referred to as asthenozoospermia, but no genes have been validated to be associated with the isolated form. However, in patients with asthenozoospermia and clinical features of primary ciliary dyskinesia (PCD), genetic testing of the PCD genes is warranted.

Asthenozoospermia is often accompanied with teratozoospermia, i. e. impaired sperm morphology. This includes sperm head defects, macrozoospermia (large sperm heads), acephalic sperm (sperm heads separated from flagella), and globozoospermia (round sperm heads/no acrosome). Further, astheno- and/or teratozoospermia are often combined with oligozoospermia [28]. A related phenotype is MMAF, which is characterised by **m**ultiple **m**orphological **a**bnormalities of the sperm **f**lagella [25]. In recent years, many associated genes have been described encoding structural components of the sperm head, midpiece, or flagellum [25] and 22 currently reach sufficient clinical validity to be incorporated into diagnostic gene panels ([23], Table 1). The genetic diagnostic yield in men with MMAF is high with 30–60 % and has clinical relevance concerning the success rates and specific procedures during MAR/ICSI [25].

### Normozoospermia and infertility

If all semen parameters are normal, the cause for couple infertility is suspected in the female partner. However, some genetic causes lead to a dysfunction of sperm that cannot be diagnosed by standard semen analysis. Indeed, defects of the sperm-specific CatSper ion channel lead to total fertilisation failure (TTF) except when using ICSI [34]. The most common underlying genetic cause with a prevalence of 2.3 % are homozygous deletions of *CATSPER2*, which can be accompanied by deletions of *STRC* resulting in variable hearing loss [34]. This contiguous gene deletion thus leads to deafness-infertility syndrome (DIS). *STRC* deletions are actually one of the most common genetic causes of hearing impairment and the ensuing CatSper defect has been an overlooked cause for couple infertility. A biochemical test has recently been developed and more and more men affected by CatSper deficiency are now identified [34]. Another example is the sperm-specific phospholipase C zeta 1 that, if impaired by variants in *PLCZ1*, also leads to fertilisation failure due to its essential role in oocyte activation [6].

**Table 1:** Selected male infertility phenotypes and associated genes. The human phenotype ontology (HPO) defines the recently improved terms for male infertility [28].

Phenotype	HPO-ID	Sperm count [concentration]	Associated genes (Mode of inheritance)
Decreased male fertility	HP:0012041		
Mild oligozoospermia	HP:0034816	30–39 million [10–15 million/mL]	to be determined
Moderate oligozoospermia	HP:0034817	10–29 million [5–10 million/mL]	to be determined
Severe oligozoospermia	HP:0034818	2–9 million [1–5 million/mL]	to be determined
Extreme oligozoospermia	HP:0034815	0–2 million [0–1 million/mL]	to be determined
Cryptozoospermia	HP:0030974	few sperm after centrifugation	Validated **crypto-/azoospermia panel** [23] *ADAD2* (AR)*, AR* (XL)*, C14orf39* (AR)*, DDX3Y* (YL)*, DMC1* (AR)*, DMRT1* (AD)*, FANCM (AR), FKBP6* (AR)*, GCNA* (XL)*, HFM1* (AR)*, KASH5* (AR)*, KCTD19* (AR)*, M1AP* (AR)*, MCM8* (AR)*, MCM9* (AR)*, MCMDC2* (AR)*, MEI1* (AR)*, MEIOB* (AR)*, MLH3* (AR)*, MSH4* (AR)*, MSH5* (AR)*, NR5A1* (AD)*, PNLDC1* (AR)*, RAD21L1* (AR)*, SHOC1* (AR)*, SPO11* (AR)*, STAG3* (AR)*, STRA8* (AR)*, SYCE1* (AR)*, TDRD9* (AR)*, TERB1* (AR)*, TERB2* (AR)*, TEX11* (XL)*, TEX14* (AR)*, TEX15* (AR)*, ZMYND15* (AR)*, ZSWIM7* (AR)
Azoospermia	HP:0000027	no sperm	
Non-obstructive azoospermia	HP:0011961	no sperm	Validated **crypto-/azoospermia panel** [23] *ADAD2* (AR)*, AR* (XL)*, C14orf39* (AR)*, DDX3Y* (YL)*, DMC1* (AR)*, DMRT1* (AD)*, FANCM (AR), FKBP6* (AR)*, GCNA* (XL)*, HFM1* (AR)*, KASH5* (AR)*, KCTD19* (AR)*, M1AP* (AR)*, MCM8* (AR)*, MCM9* (AR)*, MCMDC2* (AR)*, MEI1* (AR)*, MEIOB* (AR)*, MLH3* (AR)*, MSH4* (AR)*, MSH5* (AR)*, NR5A1* (AD)*, PNLDC1* (AR)*, RAD21L1* (AR)*, SHOC1* (AR)*, SPO11* (AR)*, STAG3* (AR)*, STRA8* (AR)*, SYCE1* (AR)*, TDRD9* (AR)*, TERB1* (AR)*, TERB2* (AR)*, TEX11* (XL)*, TEX14* (AR)*, TEX15* (AR)*, ZMYND15* (AR)*, ZSWIM7* (AR)
Spermiation failure	HP:0034812	no sperm	
Hypospermatogenesis	HP:0034813	no sperm	to be determined
Germ cell arrest	HP:0031038	no sperm	to be determined
Spermatogonial arrest	HP:4000187	no sperm	to be determined
Spermatocyte arrest	HP:0031039	no sperm	many meiotic genes from **crypto-/azoospermia panel**
Round spermatid arrest	HP:0031040	no sperm	to be determined
Sertoli cell-only	HP:0034299	no sperm	to be determined
Tubular shadows	HP:0034945	no sperm	
Obstructive azoospermia	HP:0011962	no sperm	*ADGRG2* (XL),* CFTR* (AR) [23, 29]
Congenital bilateral aplasia of the vas deferens	HP:0012873	no sperm	*ADGRG2* (XL)*,* *CFTR* (AR) [23, 29]
Asthenozoospermia	HP:0012207	reduced	*SSX1* (XL) [23]
Teratozoospermia	HP:0012864	reduced	
Sperm head anomaly	HP:0012865	reduced	*CFAP61* (AR) [23]
Globozoospermia	HP:0012205	reduced	*DPY19L2* (AR) [23]
Acephalic sperm	HP:0012869	reduced	*DNAH6* (AR), *PMFBP1* (AR)*, SUN5* (AR)*, TSGA10* (AR) [23]
Macrozoospermia	HP:0025437	reduced	*AURKC* (AR) [23]
Sperm tail anomaly	HP:0012868, HP:0033393	reduced	Validated **Multiple Morphological Abnormalities of the sperm Flagella (MMAF) panel** [23] *ARMC2* (AR)*, CFAP251* (AR)*, CFAP43* (AR)*, CFAP44* (AR)*, CFAP58* (AR)*, CFAP61* (AR)*, CFAP65* (AR)*, CFAP69* (AR)*, CFAP70* (AR)*, CFAP91* (AR)*, DNAH1* (AR)*, DNAH6* (AR)*, DNAH1* (AR)*, DNAH17* (AR)*, DNAH2* (AR)*, DNAH8* (AR)*, DNHD1* (AR)*, DRC1* (AR)*, FSIP2* (AR)*, QRICH2* (AR)*, SPEF2* (AR)*, TTC29* (AR)
Short	HP:0032559	reduced	
Bent	HP:0034811	reduced	
Coiled	HP:0032560	reduced	
Hypogonadotrophic hypogonadism	HP:0000044	reduced	Proposed **Congenital Hypogonadotrophic Hypogonadism (CHH) panel** [3, 30] *ANOS1* (XL)*, CHD7* (AD, AR, olig),* DMXL2* (AD),* DUSP6* (olig)*, FGF8* (olig)*, FGF17* (olig)*, FGFR1* (AD, AR, olig)*, FLRT3* (olig)*, GNRH1* (AR, olig)*, GNRHR* (AR, olig)*, HESX1* (AD, AR)*, IL17RD* (olig)*, KISS1* (AR)*, KISS1R* (AR)*, KLB* (AD)*, NSMF* (AR, olig)*, PNPLA6* (AR)*, POLR3A* (AR)*, POLR3B* (AR)*, PROK2* (AD, AR, olig)*, PROKR2* (AD, AR, olig)*, SEMA3A* (AD, olig)*, SEMA7A* (olig)*, SOX2* (AR)*, SOX10* (AD)*, SPRY4* (olig)*, TAC3* (AR)*, TACR3* (AR, olig)

Abbreviations: AD: autosomal dominant, AR: autosomal recessive, XL: X-linked, YL: Y-linked, olig: oligogenic

### Endocrinological causes of male infertility

Endocrine diseases can be differentiated into hypergonadotrophic and hypogonadotrophic hypogonadism both leading to inadequate spermatogenesis and, thus, reduced sperm count.

### Male hypergonadotrophic hypogonadism

Patients with genetic hypergonadotrophic hypogonadism are characterised by increased gonadotrophin levels (LH, FSH) accompanied by reduced testosterone values, small testes, and often azoospermia. In 80 % of cases, Klinefelter syndrome (karyotype 47,XXY) is the cause (see above). In the remaining 20 % of men with hypergonadotrophic hypogonadism higher-grade aneuploidies (47,XXXY), mosaicism (46,XY), or a structurally aberrant X chromosome is the underlying cause [11].

### Male hypogonadotrophic hypogonadism

Congenital hypogonadotrophic hypogonadism (CHH) can occur isolated, with anosmia (Kallmann syndrome), or in syndromic forms (e. g., Bardet-Biedl syndrome) [3]. The leading symptom of hypogonadotrophic hypogonadism is absent or arrested puberty and undervirilisation due to inadequate pituitary gonadotrophin production (typically luteinising hormone [LH] and FSH), and, thus, impaired testosterone secretion and sperm production [33]. Many monogenic causes of hypogonadotrophic hypogonadism have been described with a total diagnostic rate now reaching up to 50 % [3]. Effective hormonal treatment with gonadotrophins are available to induce spermatogenesis and may allow a spontaneous conception or MAR in couples where the male partner is affected by CHH [2].

## Tiered genetic diagnostics for female infertility

In case of couple infertility and before performing MAR treatment, the female partner has to undergo gynaecological examination. This must include anamnesis, a physical examination, an ultrasound of the inner genital tract, as well as determination of sex hormones. After exclusion of non-genetic factors, e. g. oncological diseases and associated treatments, genetic testing is indicated (Figure 1).

### Endocrine phenotypes

About 40 % of infertile women present with ovarian dysfunction accompanied by oligo- or amenorrhea. In these women, hypergonadotrophic hypogonadism, which points to primary or premature ovarian insufficiency (POI), hypogonadotrophic hypogonadism, and congenital adrenal hyperplasia need to be distinguished (Figure 1). Depending on hormonal parameters and diagnostic findings, different genetic analyses are indicated.

### Female hypergonadotrophic hypogonadism

#### Chromosomal aberrations

In women with ovarian dysfunction and after exclusion of other causes, a chromosomal analysis is indicated [17]. In 10–13 % of affected women, hypergonadotrophic hypogonadism is caused by Turner syndrome, which is a chromosomal aberration with the karyotypes 45,X, 45,X/46,XX, mosaicism, or structurally aberrant X chromosomes [30]. Women with Turner syndrome are characterised by ovarian dysgenesis and accelerated follicular atresia. Primary amenorrhea is more often observed in women with universal monosomy X, while the ovarian function and progression of puberty seem to be normal for mosaicisms of less than ~30 % of 45,X cell lines in the blood [16]. The chances for retrieval of fertilisation competent oocytes are extremely low in women with the karyotype 45,X [8]. In contrast, in cases of 45,X/46,XX mosaicism, there is an inverse correlation between the percentage of 45,X cells in the blood and the probability of a normal puberty, spontaneous cycles, and fertility [16]. Other chromosomal aberrations leading to hypergonadotrophic hypogonadism are structurally altered X chromosomes. MAR treatment for women with 45,X/46,XX mosaicism or structurally altered X chromosomes is dependent on the endocrine and clinical diagnosis.

**Table 2:** Genes associated with different forms of female infertility.

Phenotype(s)	HPO-ID	Associated genes (Mode of inheritance)
Premature/primary ovarian insufficiency (POI)	HP:0008209	Proposed **POI gene panel** [26] *BMP15* (XLD, XLR),* FANCM* (AR)*,* *FMR1* (XLD), *FOXL2* (AD, AR), *FSHR* (AR)*, GDF9* (AR)*, HFM1* (AR)*, MCM8* (AR)*, MCM9* (AR)*, MRPS22* (AR)*, NHEJ1* (AD)*, NOBOX* (AD, AR)*, NR5A1* (AD)*, PSMC3IP* (AR)*, SOHLH1* (AR)*, STAG3* (AR)
Congenital adrenal hypoplasia (CAH)	HP:0008244	*CYP21A2* (AR)* – CYP11B1* (AR)*, CYP17A1* (AR)*, HSD3B2* (AR)*, STAR* (AR)
Hypogonadotrophic hypogonadism	HP:0000044	Proposed **Congenital Hypogonadotrophic Hypogonadism (CHH) panel** [3, 30] *ANOS1* (XL)*, CHD7* (AD, AR, olig),* DMXL2* (AD),* DUSP6* (olig)*, FGF8* (olig)*, FGF17* (olig)*, FGFR1* (AD, AR, olig)*, FLRT3* (olig)*, GNRH1* (AR, olig)*, GNRHR* (AR, olig)*, HESX1* (AD, AR)*, IL17RD* (olig)*, KISS1* (AR)*, KISS1R* (AR)*, KLB* (AD)*, NSMF* (AR, olig)*, PNPLA6* (AR)*, POLR3A* (AR)*, POLR3B* (AR)*, PROK2* (AD, AR, olig)*, PROKR2* (AD, AR, olig)*, SEMA3A* (AD, olig)*, SEMA7A* (olig)*, SOX2* (AR)*, SOX10* (AD)*, SPRY4* (olig)*, TAC3* (AR)*, TACR3* (AR, olig)
Oocyte maturation arrest, fertilisation failure, zygotic cleavage defect/failure, early embryonic arrest, hydatidiform moles, recurrent pregnancy loss, multi-locus imprinting disorder	HP:0034914, not available, HP:0033336, HP:0033335, HP:0032192, HP:0200067, not available	Proposed Maternal effect genes (MEGs)* [12] *BTG4* (AR)*, CDC20* (AR)*, KHDC3L* (AD, AR)*, NLRP2* (AD, AR)*, NLRP5* (AD, AR)*, NLRP7* (AD, AR)*, PADI6* (AD, AR)*, PATL2* (AR)*, TLE6* (AR)*, TRIP13* (AR)*, TUBB8* (AD, AR)

*Variants in MEGs can cause any, some, or all of the mentioned phenotypes.

Abbreviations: AD: autosomal dominant, AR: autosomal recessive, XL: X-linked, YL: Y-linked, XLD: X-linked dominant, XLR: X-linked recessive, olig: oligogenic

Trisomy X (47,XXX) is a chromosomal aberration with variable phenotype and has been identified in 3 % of cases with POI [24]. Most women with trisomy X conceive spontaneously and show a normal endocrinological profile.

Similar to sub-/infertility in men, balanced structural chromosomal aberrations (e. g., translocation, inversions) are associated with female sub-/infertility [32]. Therefore, in case of normal clinical parameters and after exclusion of other causes, a chromosomal analysis should be performed in the male and female partner before attempting MAR. Balanced structural chromosomal aberrations regularly have no harmful effect on the carrier, but confer a higher risk to produce unbalanced gametes. This can lead either to infertility, recurrent pregnancy loss, or a birth of a child with developmental defects. Genetic counselling is important to inform about the accompanied risk for pregnancies and the offspring, and the possibility of prenatal and preimplantation genetic testing (PND/PGT).

### Monogenic causes of female infertility

About 2 % of sporadic cases with POI and 10–15 % of women with a familial predisposition carry a pre-mutation in the X-chromosomal *FMR1* [1]. The normal number of the CGG trinucleotide repeats in *FMR1* is ≤44. A pre-mutation is defined as a CGG repeat length between 55 and 200, which can increase in length when inherited from the mother. CGG repeats >200 are termed full mutation, prevent expression of *FMR1*, and brain development is impaired causing Fragile X syndrome. Therefore, the* FMR1* CGG repeat length must be analysed in women with suspected POI before MAR and women with enlarged CGG repeat segments must be offered genetic counselling.

In the recent years, exome sequencing has identified more and more genes that are associated with POI. Further, an increasing overlap of genes, mostly encoding meiosis-specific proteins, is being described to cause POI in women as well as spermatocyte/meiotic arrest and non-obstructive azoospermia in men [31]. To date no POI gene panel has been clinically validated, but a recent systematic review proposes a list of genes [26] that can serve as a suitable basis for the analysis of monogenic causes of POI (Table 2). The majority of identified variants follow an autosomal recessive inheritance. Autosomal dominant and X-linked forms are described less frequently. In addition, genes from the crypto-/azoospermia panel may also be appropriate candidate genes to be analysed in women with POI (Table 1).

### Female hypogonadotrophic hypogonadism

Similar to hypogonadotrophic hypogonadism in men, the genetic causes of CHH in women can be identified in 35–40 % of cases [17]. The isolated form of CHH can be distinguished from the Kallmann syndrome, which is accompanied by anosmia comparable to men. To verify the diagnosis, a panel analysis of CHH genes can be performed (Table 1). As in men, hormonal treatment of hypogonadotrophic hypogonadism is recommended.

### Congenital adrenal hyperplasia

Women with congenital adrenal hyperplasia (CAH) are characterised by hyperandrogenism due to increased serum androgen levels. This is a result of an impaired biogenesis of steroids in the adrenal glands, which is in more than 90–95 % of affected women caused by variants in *CYP21A2* leading to 21-hydroxylase deficiency [22]. Classic CAH, which is detected by newborn screening, is caused by biallelic severe variants, while mild forms (late onset CAH) typically are the result of biallelic variants with residual enzyme function or have been observed in women with heterozygous *CYP21A2* variants. Clinical signs are acne, hirsutism, oligo- or amenorrhea, and infertility. Thus, if such symptoms are present and before performing MAR, sequence analysis of *CYP21A2* must be performed. Variants in *CYP11B1* account with 5–8 % for the second common form of CAH [9]. Typical clinical characteristics are ambiguous genitalia in 46,XX foetuses and hyporeninemic hypokalemic hypertension. Rare forms of CAH are caused by variants in *CYP17A1* leading to delayed puberty, in *STAR* causing lipoid CAH, and in *HSD3B2* with normal sexual differentiation and/or partial virilisation in females [9, 18].

Since the carrier frequency of *CYP21A2* variants is high (1 in 60) in Middle Europe, partners of women with CAH should be informed about the option of genetic testing in order to estimate the risk of severe forms of CAH in offspring including virilisation in female foetuses. In pregnancies with at risk foetuses prenatal preventive treatment with high dose corticosteroids can be offered but is classified as experimental and should be discussed in detail [22].

### Beyond established guidelines – maternal effect genes

Maternal effect genes (MEGs) encode oocyte-specific factors that orchestrate early embryonic development prior to the onset of zygotic genome activation [12, 21]. So far, eleven MEGs have been described in humans, which are associated with a range of phenotypes that result in female infertility following an autosomal recessive or autosomal dominant inheritance (Table 2; [12]). MEGs are required during the first cell divisions, for epigenetic reprogramming and imprinting, and for the transition from maternal to embryonic developmental control. Six of the known human MEGs are components of the subcortical maternal complex (SCMC), which is an oocyte/early embryo-specific lattice structure fulfilling multiple functions. The associated phenotypes include zygotic cleavage defect/failure, early embryonic arrest, fertilisation failure, hydatidiform moles (molar pregnancy), implantation failure, oocyte maturation arrest, and recurrent pregnancy loss. Furthermore, women with pathogenic variants in MEGs are at an increased risk of having a child with multi-locus imprinting disorder (MLID) such as Beckwith-Wiedemann and Silver Russell syndrome, and of structural birth defects, such as congenital heart defects, craniofacial malformations, and defects of the neural tube [12, 13].

Most likely because of the diversity of possible phenotypes, indications to analyse MEGs are not well established and MEG-associated infertility is surely underdiagnosed. However, identifying affected women has high clinical relevance because the recurrence risk is up to 100 %. Women who might not have a chance to produce fertile oocytes may request other reproductive options such as oocyte donation, which might become available in Germany in the future [12].

The most common monogenic causes of recurrent hydatidiform moles are pathogenic variants in the MEGs *NLRP7* (55 %) and *KHDC3L* (5 %) [14, 21]. Two further MEGs are also associated with this condition: *PADI6* (1 %) and *NRPL5* (0.5 %). Additionally, three genes (*REC114, TOPVIBL, MEI1*), which play a crucial role during early meiosis, account for 0.5 % each of hydatidiform moles.

## Conclusions and outlook

Infertility is a common disease affecting one in six people. Although it is not a life-threatening condition, many affected couples experience both physical and mental distress. In many couples, MAR is one option to realise family life but personalised treatment is scarce because the underlying causes of infertility remain elusive. A thorough clinical examination of both partners is important before tiered genetic testing is indicated. Currently accepted genetic diagnostics can identify a cause in 5–10 % of women and 10–20 % of men with infertility. We expect that this percentage will increase with broader application of molecular testing strategies such as exome sequencing when these will be translated from research to clinic. A genetic diagnosis is important for medical guidance of couples with regard to treatment, the success rates for MAR, and the potential risks to their offspring. Finally, many affected persons benefit from a causal diagnosis, helping them to accept their condition and alternative courses of action.
